# Pharmacokinetics of Chiral Dendrimer-Triamine-Coordinated Gd-MRI Contrast Agents Evaluated by *in Vivo* MRI and Estimated by *in Vitro* QCM

**DOI:** 10.3390/s151229900

**Published:** 2015-12-18

**Authors:** Yuka Miyake, Syungo Ishikawa, Yu Kimura, Aoi Son, Hirohiko Imai, Tetsuya Matsuda, Hisatsugu Yamada, Akio Toshimitsu, Teruyuki Kondo

**Affiliations:** 1 Department of Energy and Hydrocarbon Chemistry, Graduate School of Engineering, Kyoto University, Nishikyo-ku, Kyoto 615-8510, Japan; miyake.yuka.46c@st.kyoto-u.ac.jp (Y.M.); m0syunc0@gmail.com (S.I.); ykimura@scl.kyoto-u.ac.jp (Y.K.); son.aoi.3n@kyoto-u.ac.jp (A.S.); yamada.hisatsugu@tokushima-u.ac.jp (H.Y.); toshi-r@iris.eonet.ne.jp (A.T.); 2 Research and Educational Unit of Leaders for Integrated Medical System, Center for the Promotion of Interdisciplinary Education and Research, Kyoto University, Nishikyo-ku, Kyoto 615-8510, Japan; 3 Graduate School of Informatics, Kyoto University, Yoshida-honmachi, Sakyo-ku, Kyoto 606-8501, Japan; imai@sys.i.kyoto-u.ac.jp (H.I.); tetsu@i.kyoto-u.ac.jp (T.M.); 4 Advanced Biomedical Engineering Research Unit, Center for the Promotion of Interdisciplinary Education and Research, Kyoto University, Nishikyo-ku, Kyoto 615-8510, Japan; 5 Division of Multidisciplinary Chemistry, Institute for Chemical Research, Kyoto University, Gokasho, Uji, Kyoto 611-0011, Japan

**Keywords:** chiral dendrimer, gadolinium, contrast agent, magnetic resonance imaging (MRI), quartz crystal microbalance (QCM), association constant (*K*_a_)

## Abstract

Recently, we developed novel chiral dendrimer-triamine-coordinated Gd-MRI contrast agents (Gd-MRI CAs), which showed longitudinal relaxivity (*r*_1_) values about four times higher than that of clinically used Gd-DTPA (Magnevist^®^, Bayer). In our continuing study of pharmacokinetic differences derived from both the chirality and generation of Gd-MRI CAs, we found that the ability of chiral dendrimer Gd-MRI CAs to circulate within the body can be directly evaluated by *in vitro* MRI (7 T). In this study, the association constants (*K*_a_) of chiral dendrimer Gd-MRI CAs to bovine serum albumin (BSA), measured and calculated with a quartz crystal microbalance (QCM) *in vitro*, were found to be an extremely easy means for evaluating the body-circulation ability of chiral dendrimer Gd-MRI CAs. The *K*_a_ values of *S*-isomeric dendrimer Gd-MRI CAs were generally greater than those of *R*-isomeric dendrimer Gd-MRI CAs, which is consistent with the results of our previous MRI study *in vivo*.

## 1. Introduction

Magnetic resonance imaging (MRI) is one of the most important non-invasive imaging modalities for disease diagnosis, and there has been dramatic progress in this field over the past 30 years [1,2,3]. Particularly, for the early and accurate diagnosis of tumors, low-molecular-weight gadolinium MRI contrast agents (Gd-MRI CAs), such as Gd-DTPA (DTPA = diethylenetriaminepentaacetic acid) and Gd-DOTA (DOTA = 1,4,7,10-tetraazacyclododecane-1,4,7,10-tetraacetic acid), are widely used clinically to obtain clear and high-contrast MR images [4,5,6,7,8,9,10]. Unfortunately, the non-specific retention and rapid clearance of low-molecular-weight Gd-MRI CAs generally require a high dosage (*ca*. 0.50 mol/L) of these Gd-MRI CAs, which imposes a great physical strain and may lead to side effects in the patient, such as osmotic shock [11,12] and nephrogenic systemic fibrosis (NSF) [13,14,15]. The main reason for such a high dosage is that up to eight of the nine coordination sites of Gd are firmly occupied by ionic chelating ligands, and only one site remains available for coordination with free water molecules, which can be observed by MRI. In addition, Gd could show fast rotational motion at the center of small ligands, and the image contrast is not high, which requires low-molecular-weight Gd-MRI CAs. Therefore, there is a strong need for the development of highly sensitive Gd-MRI CAs.

Recently, there has been growing worldwide interest in the development of highly sensitive Gd-MRI CAs that consist of Gd-functionalized dendrimers or other macromolecules [16,17,18,19]. Dendrimers [20,21,22] belong to a unique category of macromolecules with well-controlled sizes, nanoscopic dimensions, and many peripheral chemical groups to which Gd chelates can be coupled. As expected, Gd-functionalized poly(amidoamine) dendrimers (PAMAM) [23,24,25,26] and poly(propyleneimine) dendrimers (PPI) [27,28,29] have been synthesized and evaluated both *in vitro* and *in vivo* to achieve high-resolution MRI images, in which dendrimers were used as a core and Gd chelates are positioned in the periphery [30]. In the Gd-functionalized dendrimers that have been reported so far for use as MRI CAs, however, dendrimers were only used to slow the molecular tumbling and the rotation of Gd center. While the duration of intravascular retention is prolonged, the principle of reducing the ^1^H relaxation time of a water molecule is the same as that with low-molecular-weight Gd-MRI CAs.

**Figure 1:**
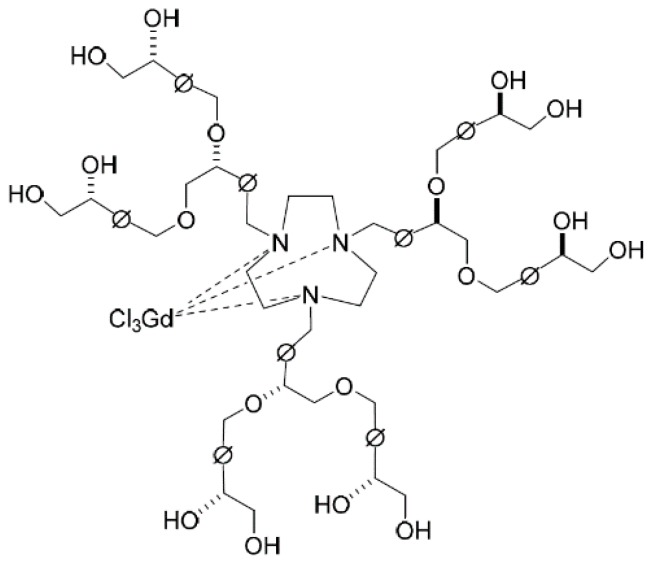
Structure of 2nd-generation chiral dendrimer-triamine-coordinated Gd-MRI CA with polyol end-groups, **2**-(*R*).

We previously reported the synthesis and functional evaluation of 2nd-generation chiral dendrimer-triamine-coordinated Gd complexes with polyol end-groups as a highly sensitive MRI CAs (Figure 1) [31,32]. More recently, we succeeded in the synthesis of chiral dendrimer-triamine-coordinated Gd-MRI CAs with aminoalcohol end-groups, and the large difference in the pharmacokinetics of the optical isomers, **2**-(*R*) and **2**-(*S*), was clarified. The synthesized Gd-MRI CAs were shown to have longitudinal relaxivity (*r*_1_) three times higher than that of clinically used Gd-DTPA. In addition, the pharmacokinetic difference between optical isomers could be easily and precisely evaluated by *T*_1_-weighted *in vivo* MRI of mice before and after the intravenous injection of **2**-(*R*) and **2**-(*S*), respectively. As a result, **2**-(*S*) is retained in the vasculature for a longer time after administration.

In this study, we found that the pharmacokinetic difference derived from both the chirality and the generation of chiral dendrimer Gd-MRI CAs can be evaluated by *in vivo* MRI and estimated by *in vitro* QCM. These results are consistent with each other. First, 2nd- and 3rd-generation chiral dendrimer-triamine-coordinated CAs, **2**-(*R*), **2**-(*S*), **3**-(*R*), and **3**-(*S*), were synthesized. As an index for evaluating the body circulation ability of these chiral dendrimer Gd-MRI CAs, association constants (*K*_a_) with plasma protein were calculated by a quartz crystal microbalance (QCM) measurement. QCM can measure a change in weight based on the fact that the resonant frequency of a quartz crystal decreases with an increase in its mass due to the adhesion of a substrate on the quartz surface [33]. While the exact amount of the substrate that adhered to the quartz surface can be calculated using the Sauebrey equation [34], the association constant (*K*_a_) of our synthesized chiral dendrimer Gd-MRI CAs with bovine serum albumin (BSA), which is a model of plasma protein, was estimated by a change in mass on the quartz surface covered with BSA, after the sequential addition of synthesized CAs.

## 2. Experimental Section

### 2.1. General Methods

All reagents were used without further purification unless otherwise noted. PPh_3_ (97.0%), *N*-bromosuccinimide (NBS, 99.9%), Ce(NH_4_)_2_(NO_3_)_6_ (CAN, 95%), GdCl_3_^.^6H_2_O, KOH (85%), K_2_CO_3_ (99.5%), MgSO_4_ (99.0%), NaHCO_3_ (99.5%), Na_2_CO_3_ (99.8%), Na_2_SO_3_ (97.0%), Na_2_SO_4_ (99.0%), CH_3_CN (99.8%), CHCl_3_ (99.9%), hexane (99%), methanol (MeOH; 99.8%), acetone (99%), tetrahydrofuran (THF), distilled water, acetic acid, and BSA were purchased from Nacalai Tesque Inc. (Kyoto, Japan). 1.0 mol/L Lithium ethoxide in tetrahydrofuran was purchased from Sigma-Aldrich Japan K. K. (Tokyo, Japan). 1,4,7-Triazacyclononane (TACN, 98%) was purchased from Tokyo Chemical Industry Co. Ltd. (Tokyo, Japan). Methylene chloride (CH_2_Cl_2_) was purchased from Kishida Chemical Co., Ltd. (Osaka, Japan). Gd-DTPA (gadopentetate meglumine; Magnevist^®^) was purchased from Bayer Holding Ltd. (Tokyo, Japan), and used as a control for all MRI measurements. Aluminum oxide 90 (activity neutral) was purchased from Merck Chemical Inc. (Darmstadt, Germany). Silica gel 60N spherical for flash chromatography was purchased from Kanto Chemical Co. Inc. (Tokyo, Japan). Filter-Aid Celite Standard Super-gel was purchased from Alfa Aesar (Lancaster, UK).

^1^H-NMR spectra were recorded at 400 MHz and ^13^C-NMR spectra were recorded at 100 MHz with a JEOL EX400 (JEOL Inc., Tokyo, Japan). ESI-TOF mass spectra were obtained by a micrOTOF focus (Bruker Daltonics Co., Billerica, MA, USA). Specific optical rotations were measured by a P-2100 polarimeter (JASCO, Tokyo, Japan). The concentration of gadolinium was determined by polarized Zeeman atomic absorption spectrometry (AAS; Z-2710, Hitachi Ltd., Tokyo, Japan) with a hollow cathode lamp (422.58 nm; L233-64NB; Hamamatsu Photonics K. K., Hamamatsu, Japan) using a gadolinium standard solution (1000 ppm, analytical grade, Wako, Japan).

### 2.2. Synthesis of 3rd-Generation Chiral Dendrimer-Triamine-Coordinated Gd-MRI CAs (Scheme 1)

#### 2.2.1. 2nd-Generation Chiral Dendron with Polyol End-Groups **6**

A solution of **5** (17.0 g, 26.0 mmol) [32] in 3 N HCl (100 mL) and CH_3_CN (200 mL) was stirred at room temperature for 4 h. The product was concentrated under vacuum, and the residue was filtered. The filtered product was washed with CH_3_CN to remove the residual volatiles. The filtrate was concentrated again under vacuum, filtered, and washed with CH_3_CN. The crude product collected was purified by recrystallization (ethyl acetate) to give **6** as a white solid (12.5 g, 45.6 mmol). ^1^H-NMR (CD_3_OD), δ (ppm): 3.52–3.60 (m, 5H); 3.68–3.71 (m, 4H); 4.34, 4.37 (d, 1H); 4.46–4.69 (m, 6H); 5.00 (s, 2H); 6.80–6.91 (m, 4H); 7.22–7.43 (m, 12H). ^13^C-NMR (CD_3_OD), *δ* (ppm): 56.1, 68.7, 71.4, 71.6, 74.0, 75.6, 75.7, 81.5, 115.7, 117.0, 127.4, 128.4, 128.8, 128.9, 138.8, 138.9, 140.1, 142.8, 154.3, 155.5. ESI-TOF-MS: *m*/*z* [M + H]^+^ calcd. for 603.7320, found 603.7352.

#### 2.2.2. 3rd-Generation Chiral Dendron with Polyacetonide End-Group **7**

To a 200 mL Pyrex flask equipped with a Dean-Stark apparatus and a reflux condenser were added **6** (2.5 g, 4.4 mmol), **5** (4.5 g, 19.7 mmol), and toluene (50 mL) in this order under a flow of argon. The mixture was heated with stirring for about 30 min to make a uniform solution. To the resulting uniform solution was added KOH pellets (2.0 g) to give a cloudy orange solution. The mixture was then heated under reflux for 12 h. After the reaction was complete (checked by TLC), the resulting solution was concentrated under vacuum, and an orange crude product with high viscosity was obtained. The crude product was dissolved in methylene chloride, and ethyl acetate and hexane were then added and suction filtration gave a uniform orange solution. The solvent was removed under vacuum, and the residue was purified by flash column chromatography (neutral Al_2_O_3_, ethyl acetate - hexane = gradient from 1:1 to 1:0) to give **7** (3.3 g, 2.5 mmol) as a yellow solid. ^1^H-NMR (CDCl_3_), δ (ppm): 1.48, 1.54 (d, 24H); 3.52–3.78 (m, 13H); 4.26–4.30 (q, 4H); 4.34–4.35 (d, 3H); 4.38–4.68 (m, 12H); 5.01–5.07(m, 6H), 6.83–6.93 (m, 4H); 7.26–7.45 (m, 28H). ^13^C-NMR (CDCl_3_), δ (ppm): 26.0, 26.6, 55.7, 70.3, 71.7, 73.0, 74.8, 77.8, 80.2, 109.7, 114.7, 115.8, 126.2, 127.1, 127.7, 127.9, 138.2, 138.3, 138.4, 138.5, 156.4, 157.3. ESI-TOF-MS: *m*/*z* [M + Na]^+^ calcd. for 1357.6440, found 1357.6463.

**Scheme 1:**
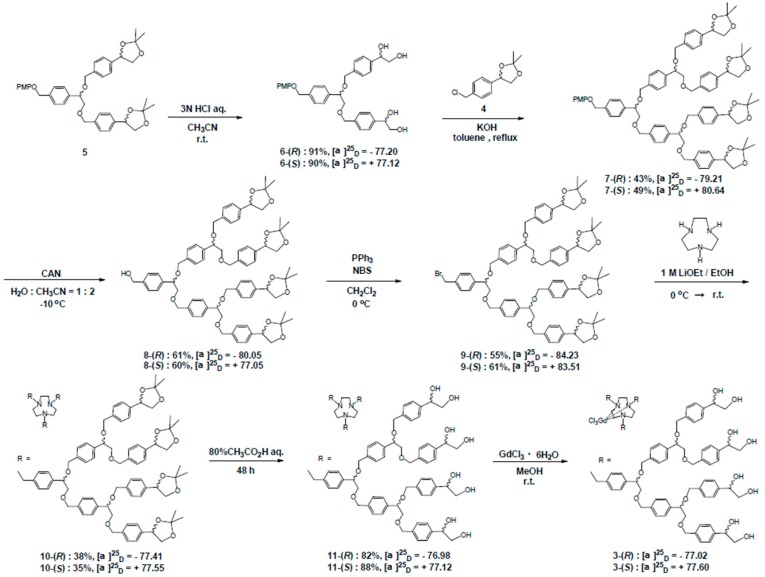
Synthesis of 3rd-generation chiral dendrimer-triamine-coordinated Gd complexes, **3**-(*R*) and **3**-(*S*).

#### 2.2.3. 3rd-Generation Chiral Dendron **8** from **7** by Deprotection

A solution of **7** (1.47 g, 1.05 mmol) in CH_3_CN (50 mL) was stirred at −10 °C, and a solution of CAN (1.15 g, 2.10 mmol) in water (25 mL) was added dropwise. After the addition was complete, the resulting mixture was stirred for 30 min and then cooled to 0 °C, followed by the addition of water (150 mL). The resulting mixture was transferred to a separating funnel, and after extraction with ethyl acetate and water, the water layer was extracted three times with ethyl acetate. The organic layer was collected and washed twice with a 5% K_2_CO_3_ aqueous solution. The organic layer was combined and washed with a 10% Na_2_SO_3_ aqueous solution and 5% K_2_CO_3_ aqueous solution. The organic layers were then washed with brine and dried over MgSO_4_, followed by concentration under vacuum. The crude product was purified by column chromatography (SiO_2_, ethyl acetate-hexane = gradient from 1:1 to 100:0) to give **8** (1.028 g, 837 µmol, 80%) as an orange solid. ^1^H-NMR (CDCl_3_), δ (ppm): 1.48, 1.54 (d, 24H); 3.52–3.80 (m, 10H); 4.26–4.28 (m, 4H); 4.30–4.68 (m, 17H); 5.04–5.07 (m, 4H); 7.26–7.36 (m, 28H). ^13^C-NMR (CDCl_3_), δ (ppm): 26.0, 26.6, 65.0, 70.3, 71.6, 73.0, 74.8, 77.8, 80.2, 109.7, 126.2, 127.1, 127.7, 127.9, 138.2, 138.3, 138.4, 138.5. ESI TOF MS: *m*/*z* [M + Na]^+^ calcd. for 1251.6021, found 1251.5840.

#### 2.2.4. 3rd-Generation Chiral Dendron **9** from **8** by Bromination

**8** (1.03 g, 837 µmol) and methylene chloride (20 mL) were added to a 100 mL Pyrex flask under a flow of argon. The flask was immersed in a cooling bath, and at 0 °C, PPh_3_ (235 mg, 902 µmol) and NBS (170 mg, 955 µmol) were added together under a flow of argon. The mixture was stirred for 2 h at 0 °C. After the reaction was complete (checked by TLC), to the reaction mixture were added diethyl ether (10 mL) and saturated Na_2_CO_3_ aqueous solution (10 mL). After the solution was separated, the organic layer was washed with brine and dried over MgSO_4_. The resulting organic layer was concentrated under vacuum, and the residue was purified by column chromatography (SiO_2_, ethyl acetate-hexane = gradient from 3:1 to 1:1) to give **9** as a white solid (320 mg, 248 µmol, 30%). ^1^H-NMR (CDCl_3_), δ (ppm): 1.47, 1.54 (d, 24H); 3.52–3.73 (m, 10H); 4.25–4.29 (m, 4H); 4.32–4.71 (m, 17H); 5.02–5.07 (m, 4H); 7.22–7.38 (m, 28H). ^13^C-NMR (CDCl_3_), δ (ppm): 25.8, 26.4, 33.1, 67.7, 70.3, 71.4, 72.8, 74.2, 77.6, 80.0, 109.5, 125.9, 126.1, 127.3, 127.7, 138.2, 138.3, 138.4, 138.5. ESI-TOF-MS: *m*/*z* [M + H]^+^ calcd. for 1313.5177, found 1313.5186.

#### 2.2.5. 3rd-Generation Chiral Dendrimer with Polyacetonide End-Group **10**

A solution of LiOEt in THF (1.0 mol/L, 5.0 mL) was added dropwise to a solution of TACN (10.0 mg, 80.0 µmol) in THF (20 mL), and the mixture was stirred at 0 °C under an argon atmosphere. After 10 min, **9** (320 mg, 247 µmol) was added dropwise at 0 °C, and the mixture was stirred at room temperature for 12 h. The mixture was then diluted by the addition of ethyl acetate (40 mL), and dried over MgSO_4_. The solvent was removed under reduced pressure, and the residue was purified by flash column chromatography (SiO_2_, CHCl_3_:MeOH = 20:1) to give 65.0 mg (17.3 µmol, 22%) of **10**. ^1^H-NMR (CDCl_3_), δ (ppm): 1.40, 1.46 (s, 72H); 2.77 (br, 12H); 3.44–3.66 (m, 36H); 4.18–4.22 (t, 12H); 4.27–4.59 (m, 45H); 4.96–4.99 (t, 12H); 7.18–7.24 (m, 84H). ^13^C-NMR (CDCl_3_), δ (ppm): 25.9, 26.6, 55.0, 70.3, 71.6, 73.0, 73.2, 74.8, 77.7, 80.4, 109.6, 126.2, 127.1, 127.7, 127.8, 138.1, 138.2, 138.3, 138.4. ESI-TOF-MS: *m*/*z* [M + H]^+^ calcd. for 3764.7120, found 3764.8410.

#### 2.2.6. 3rd-Generation Chiral Dendrimer with Polyol End-Group **11**

**10** (60.0 mg, 16 µmol) was dissolved and stirred in 80% acetic acid/water (5.0 mL) at room temperature for 6 h, the reaction was monitored by ESI-TOF MS. After the reaction was complete, the solvent was removed under reduced pressure to give 42 mg (13 µmol, 81%) of **11**. ^1^H-NMR (CD_3_OD), δ (ppm): 2.82 (br, 12H); 3.54–3.72 (m, 36H); 4.31–4.44 (m, 57H); 4.62–4.64 (m, 12H); 7.14–7.27 (m, 84H). ^13^C-NMR (CD_3_OD), δ (ppm): 50.2, 60.6, 68.7, 71.8, 74.0, 75.3, 75.6, 81.4, 127.4, 128.6, 128.7, 131.6, 136.1, 138.7, 141.6, 142.7. ESI-TOF-MS: *m*/*z* [M + H]^+^ calcd. for 3283.9320, found 3283.9919.

#### 2.2.7. 3rd-Generation Chiral Dendrimer-Triamine-Coordinated Gadolinium Complex **3**

A solution of GdCl_3_·6H_2_O (4.8 mg, 12.9 µmol) in MeOH (5.0 mL) was added dropwise to a solution of **11** (42.0 mg, 12.8 µmol) in MeOH (5.0 mL), and the mixture was stirred at room temperature for 24 h. The solvent was removed under reduced pressure to give 45.0 mg (12.7 µmol) of **3** as a white solid.

### 2.3. Relaxation Time Measurements

Longitudinal relaxivity (*r*_1_) was calculated from the *T*_1_-relaxation time of water proton measured under four different concentrations (0.50, 0.25, 0.10, 0 mmol/L) of Gd-MRI CAs at 20 °C with a Biospec 7.0 T/20 USR (Bruker Biospin Inc., Billerica, MA, USA) and a 72 mm i.d. Quadrature resonator.

### 2.4. Cytotoxicity Assay

Murine L929 fibroblast cells were placed in each well of 96-multiwell cell culture plates (Corning Inc., Lowell, MA, USA) with growth medium (Dulbecco’s modified minimum essential medium (DMEM); Life Technologies Japan Ltd., Tokyo, Japan) containing 10% of fetal bovine serum (FBS; Sanko Pure Chemical Co., Tokyo, Japan), 100 mg/mL of penicillin and 0.10 mg/mL of streptomycin (Sigma-Aldrich Japan K.K., Tokyo, Japan) at a density of 1 × 10^4^ cells/cm^2^. After incubation for 24 h at 37 °C and 5% CO_2_/95% air at atmospheric pressure, the medium was replaced by fresh growth medium containing 0.25 mmol/L of Gd-MRI CAs. After incubation for 48 h under the same conditions, the cell number was evaluated. After Cell Count Reagent SF (10 µL, Nacalai Tesque, Kyoto, Japan) was added to each well, plates were incubated for 60 min under the same conditions. The absorbance at 450 nm was then measured by a spectrophotometer (Versa max, Molecular Devices Inc., Union City, CA, USA). The absorbance was normalized by that in cells incubated with growth medium without Gd-MRI CAs and expressed as a cell viability ratio.

### 2.5. Quartz Crystal Microbalance (Affinix-Q^®^) Analysis

The ability of Gd-MRI CAs to interact with bovine serum albumin (BSA) as a model protein of blood plasma was evaluated with a quartz crystal microbalance system (Affinix-Q^®^, Initium Inc., Tokyo, Japan). Briefly, 5 µL of a mixture of H_2_SO_4_ and H_2_O_2_ (3:1, called as piranha solution) was mounted to the Au surface (5.7 mm^2^) on the quartz crystal sensor, and incubated for 5 min at room temperature. The sensor was then washed thoroughly with double-distilled water, and the washing was repeated three times. After the sensor was dried, the frequency of the quartz sensor was recorded in air and in water at 25 °C with stirring at 600 rpm, where the drift of the sensor was kept below 3.0 Hz/min. Next, 2.0 µL of BSA solution (100 µg/mL in water) was applied to the sensor and BSA immobilization was performed for 15 min with incubation at room temperature under saturated humidity. After the immobilized-BSA sensor was washed with water three times, it was immersed in a batch cell with 8.0 mL water. The amount of immobilized-BSA was calculated from the change in frequency before and after immobilization. A corresponding amount of sample was dissolved with 8.0 µL of water, and injected into the batch cell. Changes in frequency were recorded point by point until the adsorption was saturated. The dissociation rate constant (*K*_d_) was estimated using AQUA software (Initium Inc., Tokyo, Japan) by a global fitting analysis of the saturated adsorption amount at each concentration, and the association rate constant (*K*_a_) was calculated as 1/*K*_d_.

### 2.6. Evaluation of the Body Distribution of Chiral Dendrimer Gd-MRI CAs by in Vivo MRI

All the animal experiments were performed according to the Institutional Guidelines of Kyoto University on Animal Experimentation and with permission from the Animal Experiment Committee of the Faculty of Medicine, Kyoto University. Chiral dendrimer Gd-MRI CAs were injected into the tail vein of ddY mice (6 or 8 weeks old, Shimizu Laboratory Supplies, Co. Ltd., Kyoto, Japan) intravenously. The time course of distribution of chiral dendrimer Gd-MRI CAs in a mouse body was evaluated by obtaining *T*_1_-weighted MR images before and after injection. To evaluate the pharmacokinetics of chiral dendrimer Gd-MRI CAs, mice were laparotomized at 30 min and 60 min after the administration of chiral dendrimer Gd-MRI CAs (0.10 mmol Gd/kg) to collect blood from the heart, and the concentration of gadolinium in blood was determined with AAS (*n* = 3). The total amount of chiral dendrimer Gd-MRI CAs in blood was estimated from the volume of total blood (78.0 mL/kg). After blood collection, the liver and kidney of the mice were minced with scissors, and gadolinium in the tissues was extracted with 5.0 mL of 1.0 mol/L HNO_3_ aq. After filtration (Millipore Millex^®^ LG 0.20 μm; Merck KGaA, Darmstadt, Germany), the gadolinium concentration in these filtrates was determined with AAS, and the total amount of chiral dendrimer Gd-MRI CAs in liver and kidney was estimated from the weight of the tissues. In addition, urine was collected from mice at 60 min after the injection of chiral dendrimer Gd-MRI CAs, and the gadolinium content was determined by AAS after dilution with 1.0 mol/L HNO_3_ aq. and filtration (*n* = 3; each sample was gathered from three mice).

## 3. Results and Discussion

### 3.1. Synthesis of 2nd- and 3rd-Generation Chiral Dendrimer-Triamine-Coordinated Gd-MRI CAs

2nd-generation chiral dendrimer-triamine-coordinated Gd complexes, **2**-(*R*) and **2**-(*S*), were synthesized according to our reported method [31]. As shown in Scheme 1, 3rd-generation chiral dendrimer-triamine-coordinated Gd complexes, **3**-(*R*) and **3**-(*S*), were synthesized from **5** through the repeated protection/deprotection of chiral 1,2-diol groups and a benzylic bromide to be connected to a triamine core.

### 3.2. Functional Evaluation of Chiral Dendrimer-Triamine-Coordinated Gd Complexes, **2**-(R), **2**-(S), **3**-(R), and **3**-(S), as Highly Sensitive MRI Contrast Agents

#### 3.2.1. Functional Evaluation of Chiral Dendrimer Gd Complexes by *in Vitro* MRI

**Figure 2:**
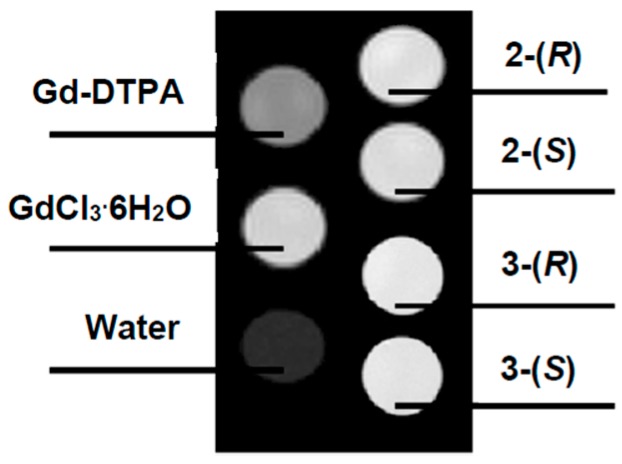
*T*_1_-weighted MR images of Gd-DTPA, GdCl_3_^.^6H_2_O, **2**-(*R*), **2**-(*S*), **3**-(*R*), **3**-(*S*), (0.25 mmol/L) and water (Spin echo from TR/TE 200/6.2 ms, 7 T, 20 °C).

The longitudinal relaxivity values (*r*_1_) of new chiral dendrimer Gd complexes as well as Gd-DTPA were measured (Figure 2, Table 1). The *r*_1_ values of **2**-(*R*) and **2**-(*S*) (2nd-generation) were 11.4 and 11.1 (mmol/L)^−1^·s^−1^, respectively, which were approximately 2.5 times larger than that of the clinically used Gd-DTPA. In addition, the *r*_1_ values of **3**-(*R*) and **3**-(*S*) (3rd-generation) were 16.3 and 16.4 (mmol/L)^−1^·s^−1^, respectively, which were approximately 3.6 times larger than that of Gd-DTPA. These results indicate that the *r*_1_ values of the Gd complexes increased as the size and the generation of chiral dendrimer ligands increased (Table 1). This can be explained by both the approach and coordination of many water molecules to a gadolinium center through the hydrogen bonding with polyol end-groups in chiral dendrimer Gd complexes, which are more prevalent in bigger dendrimers. Consequently, all of these chiral dendrimer Gd complexes, **2**-(*R*), **2**-(*S*), **3**-(*R*), and **3**-(*S*), are highly efficient MRI contrast agents.

**Table 1:** Longitudinal relaxivities (*r*_1_) of Gd-DTPA, **2-**(*R*), **2-**(*S*), **3**-(*R*) and **3**-(*S*).

Sample	*r*_1_ ((mmol/L)^−1^·s^−1^)
**Gd-DTPA**	4.6
**2-**(*R*)	11.4
**2-**(*S*)	11.1
**3-**(*R*)	16.3
**3-**(*S*)	16.4

#### 3.2.2. Cytotoxicity of Chiral Dendrimer Gd-MRI CAs

**Figure 3:**
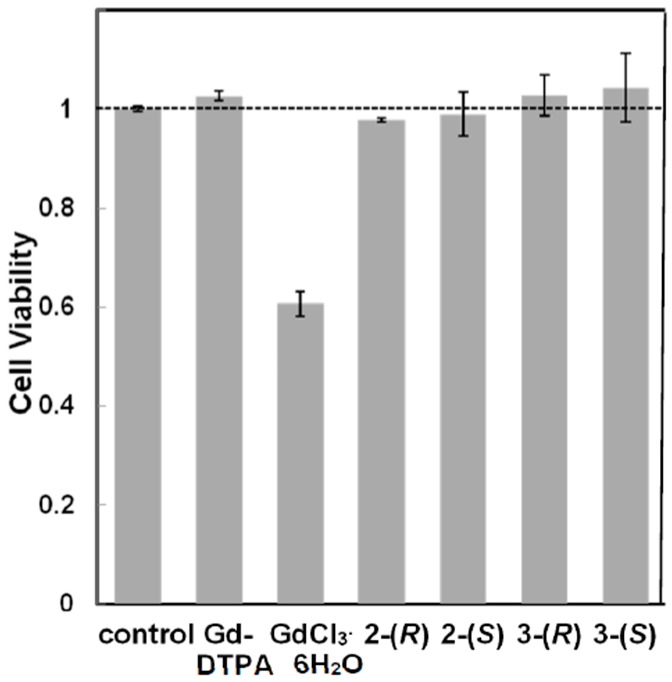
Viabilities of L929 cells 24 h after incubation with Gd-DTPA, GdCl_3_^.^6H_2_O, **2**-(*R*), **2**-(*S*), **3**-(*R*) and **3**-(*S*) (0.25 mmol/L).

The cytotoxicities of **2**-(*R*), **2**-(*S*), **3**-(*R*), **3**-(*S*), GdCl_3_·6H_2_O, and Gd-DTPA are summarized in Figure 3. There were no obvious differences in cell viability between chiral dendrimer Gd-MRI CAs and Gd-DTPA, even at a high concentration of up to 0.25 mmol/L. Only GdCl_3_·6H_2_O showed cytotoxicity at 0.25 mmol/L. Thus, chiral dendrimer Gd-MRI CAs have low toxicity with excellent size- and molecular-weight-dependent sensitivity, suitable for obtaining high-contrast MR images.

#### 3.2.3. Functional Evaluation and Pharmacokinetics of Chiral Dendrimer Gd-MRI CAs by *in Vivo* MRI

**Figure 4:**
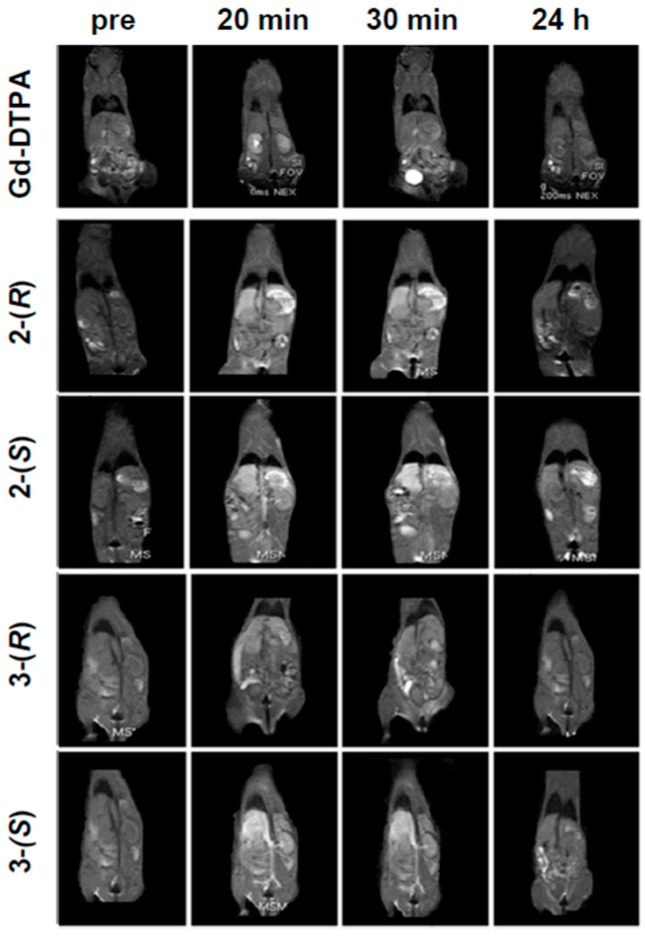
*T*_1_-weighted MR images of mice after intravenous injection of Gd-DTPA, **2**-(*R*), **2**-(*S*), **3**-(*R*) and **3-**(*S*) (0.10 mmol Gd/kg).

Contrast enhancement after the administration of chiral dendrimer Gd-MRI CAs (**2**-(*R*), **2**-(*S*), **3**-(*R*), and **3**-(*S*)) as well as Gd-DTPA was evaluated by *in vivo* MRI. Figure 4 shows *T*_1_-weighted MR images of mice before and after intravenous injection of the Gd-MRI CAs, where the dose (0.10 mmol Gd/kg) was determined with reference to that of clinically used Gd-DTPA.

After the injection of Gd-DTPA, most Gd-DTPA was excreted through the kidneys within 30 min, and accumulated in the bladder, while little contrast enhancement was observed except for the kidneys. In sharp contrast, after the injection of 2nd-generation chiral dendrimer Gd-MRI CAs, **2**-(*R*) and **2**-(*S*), no accumulation was observed in specific organs such as the liver and kidney. In comparison to Gd-DTPA, **2**-(*R*) and **2**-(*S*) should be suitable for magnetic resonance angiography (MRA) because they are retained in the blood for a long duration. There was no obvious difference in the body distributions of **2**-(*R*) and **2**-(*S*).

Similarly, after the injection of **3**-(*R*) and **3**-(*S*), no accumulation was observed in specific organs such as the liver and kidney. The most important difference between **3**-(*R*) and **3**-(*S*) was that **3-**(*S*) provided greater contrast enhancement than **3-**(*R*), when evaluated 30 min after injection. In addition, **3**-(*R*) and **3**-(*S*) provided much greater contrast enhancement than **2**-(*R*) and **2**-(*S*), which means that the difference in contrast enhancement derived from the chirality in dendrimer ligands is highly dependent on both the size and generation of chiral dendrimer ligands, judging from the *r*_1_ values (*vide supra*). No gadolinium accumulation was observed in the liver, kidney, or blood vessels within 48 h after the injection of the chiral dendrimer Gd-MRI CAs by AAS, which indicates that all of the chiral dendrimer Gd-MRI CAs were completely excreted from the body.

**Figure 5:**
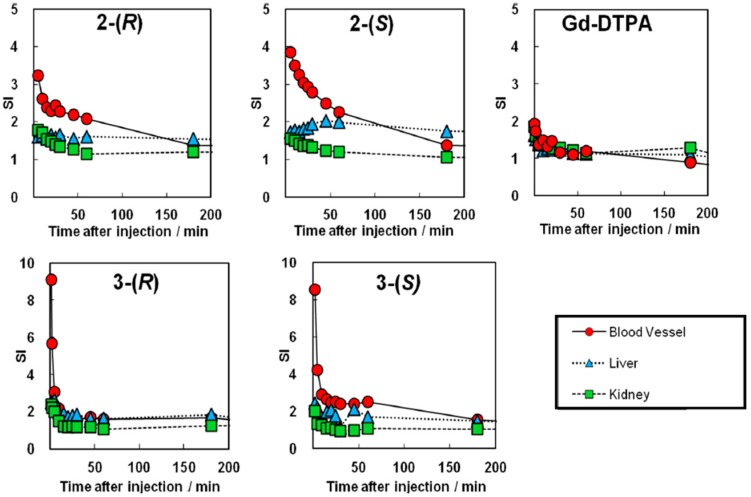
Post/pre signal intensity (SI) enhancement of mouse tissues after intravenous injection of Gd-DTPA, **2**-(*R*), **2**-(*S*), **3**-(*R*) and **3**-(*S*) (0.10 mmol Gd/kg).

The signal intensities of blood vessels and the distributions of all chiral dendrimer Gd-MRI CAs in organs up to 3 h after administration were calculated based on the results shown in Figure 4. In addition, Figure 5 shows that the retention times of all of the new chiral dendrimer Gd-MRI CAs were greater than that of Gd-DTPA.

**2**-(*S*) is retained in blood vessels somewhat longer than **2**-(*R*). Thus, **2**-(*S*) would be superior to **2**-(*R*) in the circulation of a mouse without accumulation in specific organs, and should be suitable for detecting cancer tissues that contain many new blood vessels. The slight enhancement of contrast in the liver also supports this possibility since **2**-(*S*) may show superior circulation in the liver which contains many blood vessels (Figure 4).

As for 3rd-generation Gd-MRI CAs, the calculated signal intensities of blood vessels suggest that **3**-(*S*) is retained in the blood vessels longer than **3**-(*R*) (Figure 5). This means that **3**-(*S*), rather than **3**-(*R*), would have a long circulation time in mice without accumulation in specific organs, and would be suitable for detecting cancer tissues that contain many new blood vessels.

**Figure 6:**
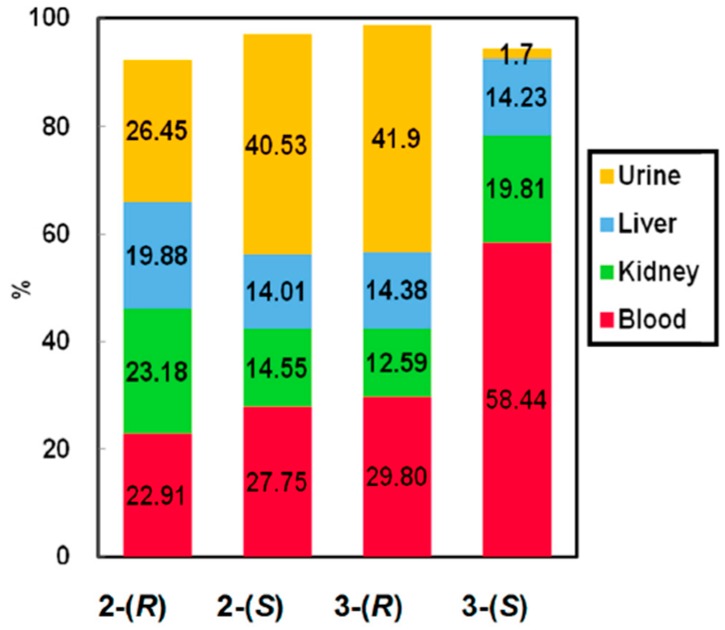
Body distribution of **2**-(*R*), **2**-(*S*), **3**-(*R*) and **3**-(*S*) 60 min after intravenous injection into mice (0.10 mmol Gd/kg).

The urine of mice was collected after the administration of chiral dendrimer Gd-MRI CAs (**2**-(*R*) and **2**-(*S*), **3**-(*R*), and **3**-(*S*)), and the gadolinium contents in urine were quantified by AAS. In addition, the mice were sacrificed in 60 min after the administration, and the liver, kidney and blood were collected and the concentrations of gadolinium in these organs and blood were analyzed by AAS. Figure 6 shows the distribution of gadolinium in urine, liver, and blood at 60 min after the administration of the new CAs. As can be readily seen from Figure 6, all of the new chiral dendrimer Gd-MRI CAs were traced quantitatively, but there was no significant difference in the pharmacokinetics of **2**-(*R*) and **2**-(*S*) at 60 min after injection.

In addition, cumulative quantification of Gd^3+^ in urine by AAS clearly showed that Gd^3+^ in **2**-(*R*) and **2**-(*S*) was completely excreted, respectively, without accumulation in the tissues and organs at 24 h after injection. If the chiral dendrimer-triamine ligands in **2**-(*R*) and **2**-(*S*) dissociate from Gd^3+^ (the circulating concentration of Gd^3^^+^ was estimated as *ca*. 1.27 mmol/L) and are transferred to free metals with coordination in the blood, such as Ca^2+^ (0.65 mM) and Mg^2+^ (0.40 mmol/L) [35], the free Gd^3+^ without ligands easily forms GdPO_4_ which accumulates and remains in the bone, kidney, and liver. Accordingly, we consider that all novel chiral dendrimer-triamine-coordinated Gd CAs have enough stability to survive even in the blood containing free metal ions [36]. The cumulative assay of gadolinium in mouse urine after the administration of **3**-(*R*) and **3**-(*S*) clearly shows that **3**-(*R*) is excreted in the urine faster than **3**-(*S*).

#### 3.2.4. Affinity of Chiral Dendrimer Gd-MRI CAs with Plasma Protein Estimated by *in Vitro* Quartz Crystal Microbalance Measurement

To estimate the difference in pharmacokinetics of chiral dendrimer Gd-MRI CAs, the interaction and affinity of chiral dendrimer Gd-MRI CAs with plasma proteins was measured by a quartz crystal microbalance (QCM) method. QCM makes it possible to measure a change in weight based on the fact that the resonant frequency of a quartz crystal decreases with an increase in its mass, which results from the adhesion of some substance to the quartz surface. The association constants (*K*_a_) of chiral dendrimer Gd-MRI CAs with bovine serum albumin (BSA), which is a model of plasma proteins, were estimated from the change in mass for a quartz surface that was first covered with BSA and then subjected to the sequential addition of chiral dendrimer Gd-MRI CAs. From the change in mass (Δ*F*) and concentrations of added chiral dendrimer Gd-MRI CAs ([CA]), the change in frequency under the saturated adhesion of chiral dendrimer Gd-MRI CAs (Δ*F*_max_) can be estimated through a least squares approximation using Equation. The concentration of [CA] that would give half of Δ*F*_max_ under a change in mass could be assumed to be *K*_a_^−1^:

$$\Delta F_{t\to \infty }=\frac{\Delta F_{max}{\left[CA\right]}_{0}}{K_{a}^{-1}+{\left[CA\right]}_{0}}$$

**Figure 7:**
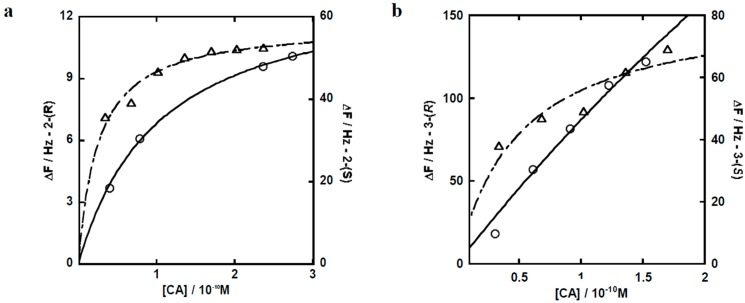
Frequency change of the Au electrodes on quartz crystals coated with BSA after addition of (**a**) **2**-(*R*) (○) and **2**-(*S*) (△); (**b**) **3**-(*R*) (○) and **3**-(*S*) (△).

Based on the results in Figure 7 and Table 2, **2**-(*S*) showed a *K*_a_ value that was about 57-fold higher than that of **2**-(*R*). This means that **2**-(*S*) is retained longer in the blood than **2**-(*R*). Also, **3**-(*S*) showed about a *K*_a_ value that was about 19-fold higher than that of **3**-(*R*). Although Δ*F* of **3**-(*R*) appeared to be greater than that of **3**-(*S*) in Figure 7b, the *K*_a_ of **3**-(*S*) was estimated to be higher than that of **3**-(*R*) by an approximation curve (Equation (1)). This means that **3**-(*S*) is retained longer in the blood than **3**-(*R*). In addition, the reason why a large difference in *K*_a_ was observed for 3rd-generation CAs (**3**-(*R*) and **3**-(*S*)), but not 2nd-generation CAs (**2**-(*R*) and **2**-(*S*)), is considered to be the increase in the number of chiral centers, which leads to an increase in optical rotation. In fact, the number of chiral centers in chiral dendrimer Gd-MRI CAs was increased from 9 (**2**-(*R*) and **2**-(*S*)) to 21 (**3**-(*R*) and **3**-(*S*)).

**Table 2:** Association constant (*K*_a_) of synthesized CAs (**2**-(*R*), **2**-(*S*), **3**-(*R*) and **3**-(*S*)) with bovine serum albumin (BSA).

Sample	*K_d_* (mol/L)	*K_a_* (mol/L^−1^)	Δ*F_max_*
**2-**(*R*)	1.42 × 10^−9^	7.04 × 10^8^	13.7
**2-**(*S*)	2.49 × 10^−11^	4.02 × 10^10^	58.3
**3-**(*R*)	1.02 × 10^−9^	9.80 × 10^8^	969
**3-**(*S*)	5.57 × 10^−11^	1.80 × 10^10^	83.8

Generally, drugs (including CAs) that are administered in blood strongly interact with plasma proteins through association and dissociation processes, until an equilibrium is reached. On glomerular filtration, which is one of the most important metabolic pathways, only CAs in a dissociated state can be excreted. Accordingly, the difference in the affinity of chiral dendrimer Gd-MRI CAs for plasma proteins affects their metabolism, and could result in differences in body distribution, *i.e.*, pharmacokinetics. In this study, the evaluation of CAs by *in vivo* MRI was consistent with the estimation of CAs by *in vitro* QCM, and QCM offers a highly useful and precise method for investigating the biocompatibility of CAs *in vitro*.

## 4. Conclusions/Outlook

In conclusion, novel chiral dendrimer-triamine-coordinated 2nd- and 3rd-generation Gd-MRI CAs, **2-**(*R*), **2-**(*S*), **3-**(*R*), and **3-**(*S*), were synthesized with high optical purity. The pharmacokinetics of these chiral dendrimer Gd-MRI CAs were then evaluated by *in vivo* MRI and estimated by *in vitro* QCM.

As a result, the *r*_1_ values of chiral dendrimer Gd-MRI CAs were almost 4 times higher than that of clinically used Gd-DTPA. None of the chiral dendrimer Gd-MRI CAs showed any cytotoxicity at a concentration of 0.25 mmol/L. By *in vivo* MRI, all of the chiral dendrimer Gd-MRI CAs circulated in a mouse body for a long time without accumulation in specific organs, and **3-**(*S*) was retained longer in the blood than **3**-(*R*). These results correspond to the affinity of these compounds for plasma proteins such as BSA, as shown by QCM measurements.

Consequently, the size and generation of dendrimer Gd-MRI CAs strongly affect their retention in blood and circulation in the body, while the chirality of dendrimer Gd-MRI CAs influenced their affinity for plasma proteins such as BSA, as shown by *in vitro* QCM measurements. The present study, which combined the use of MRI with QCM, could open doors to the synthesis, analysis, and application of novel chiral dendrimer Gd-MRI CAs with high resolution and performance. Hopefully, the present study can also help to reduce the dose of Gd-MRI CAs, which could improve patients’ quality of life (QOL).
